# Comparison of non-criteria antiphospholipid syndrome with definite antiphospholipid syndrome: A systematic review

**DOI:** 10.3389/fimmu.2022.967178

**Published:** 2022-08-18

**Authors:** Gilberto Pires da Rosa, Ester Ferreira, Bernardo Sousa-Pinto, Ignasi Rodríguez-Pintó, Iva Brito, Alberto Mota, Ricard Cervera, Gerard Espinosa

**Affiliations:** ^1^ Department of Autoimmune Diseases, Hospital Clínic, Institut d’Investigacions Biomèdiques August Pi I Sunyer (IDIBAPS), University of Barcelona, Barcelona, Spain; ^2^ Department of Dermatovenereology, Centro Hospitalar Universitário de São João, Porto, Portugal; ^3^ Faculty of Medicine, University of Porto, Porto, Portugal; ^4^ Department of Internal Medicine, Centro Hospitalar Universitário de São João, Porto, Portugal; ^5^ MEDCIDS – Department of Community Medicine, Information and Health Decision Sciences, Faculty of Medicine, University of Porto, Porto, Portugal; ^6^ CINTESIS – Center for Health Technology and Services Research, Porto, Portugal; ^7^ Autoimmune Diseases Unit, Hospital Universitari Mútua de Terrassa, Terrassa, Spain; ^8^ Department of Rheumatology, Centro Hospitalar Universitário de São João, Porto, Portugal; ^9^ Department of Medicine, Faculty of Medicine, University of Porto, Porto, Portugal

**Keywords:** antiphospholipid syndrome, treatment, clinical manifestations, non-criteria, seronegative, probable, low titre, antiphospholipid antibodies

## Abstract

**Objectives:**

Patients with laboratory or clinical manifestations suggestive of antiphospholipid syndrome (APS) but not fulfilling the classification criteria constitute a clinical challenge. This study aims to compare non-criteria APS (NC-APS) with definite APS in terms of clinical manifestations, therapies, and outcomes.

**Methods:**

A systematic review of observational studies comparing definite and NC-APS was performed searching four electronic databases. Data on clinical manifestations, therapies and clinical outcomes was extracted.

**Results:**

Sixteen studies, assessing a total of 3,798 participants, were included. Seven out of 10 studies found no significant difference in the prevalence of arterial or venous thrombosis between definite and NC-APS, with two studies on seronegative APS also finding no difference in thrombosis recurrence. Seven out of 12 studies found no significant difference in the prevalence of obstetric manifestations between groups, with the remaining exhibiting conflicting results. In 9 studies comparing treatment frequency in obstetric patients, all but one described similar treatment frequency, with the percentage of NC-APS treated during pregnancy ranging from 26% to 100%. In 10 studies comparing pregnancy outcomes of NC-APS versus definite APS, 7 found similar successful pregnancies/live births. Additionally, 5 studies described improvement of live births in both groups with treatment, with three signalling aspirin monotherapy as efficacious as combination therapy in NC-APS.

**Conclusion:**

This review hints at an absence of marked differences in most evaluated parameters between definite and NC-APS, emphasizing the value of a more active follow-up of these patients. The low-quality available evidence highlights the need for well-defined NC-APS populations in future studies.

**Systematic Review Registration:**

https://www.crd.york.ac.uk/prospero, identifier CRD42020210674.

## Introduction

Patients with laboratory or clinical manifestations suggestive of antiphospholipid syndrome (APS) but not fulfilling the Sydney Classification Criteria for definite APS (1) constitute a relevant challenge in clinical practice. Since the description of seronegative APS (SN-APS) by Hughes and Khamashta in 2003 (2), various publications have discussed the existence and characteristics of these patients (3–6). Furthermore, other elements not included in the classification criteria drew attention, namely “non-criteria” clinical manifestations (1, 5, 7–9). The report of the *14th International Congress on Antiphospholipid Antibodies Technical Task Force on APS clinical manifestations* highlighted the role of some of these features on the clinical course of the disease (5). Accordingly, efforts for the development of new classification criteria are currently underway, with the prospect of the eventual inclusion of some of these manifestations (10).

From a laboratory perspective, a growing number of publications have suggested that a larger number of patients would be classified as APS if the array of antibodies tested was expanded to include non-criteria antiphospholipid antibodies (aPL) (3, 11–16). Finally, even the current laboratory criteria raise questions, namely in the matter of the relevance of low titres of anticardiolipin (aCL) and anti-β2 glycoprotein I (anti-β2GPI) antibodies (17–21).

Consequently, there is a substantial number of patients who fit the profile of “non-criteria” APS (NC-APS) and a rising number of studies that include these patients; however, the small samples and the different definitions of non-criteria patients greatly undermine the formulation of generalizable conclusions (5, 22, 23). We recently elaborated a nomenclature proposal for research purposes (24) and categorized patients who do not fulfil the classification criteria in four subsets, under the broad term NC-APS. A review of the available data can enable a deeper understanding of their clinical characteristics and prognosis in comparison with patients with definite APS. Additionally, analysing potential subsets of “non-criteria” APS can help clarify discrepancies and similarities among them and suggest possible management specificities.

Therefore, we performed a systematic review of studies comparing patients with NC-APS with patients with definite APS in terms of the frequency of clinical manifestations (vascular thrombosis and pregnancy morbidity), prescribed therapies and reported clinical outcomes.

## Methods

The systematic review protocol was registered with the International Prospective Register of Systematic Reviews (PROSPERO) on October 24, 2020, with registration number CRD42020210674.

### Eligibility criteria

We included retrospective cohort studies comparing participants with NC-APS with those with definite APS regarding their clinical manifestations, therapies, and outcomes. The term NC-APS was considered to include patients with clinical and/or laboratory manifestations suggestive of APS but not fulfilling the Sydney Classification Criteria for Definite APS (1). Case reports and papers focusing exclusively on paediatric populations were excluded, as the disease is understudied and carries certain specificities in this age group (e.g., the absence of obstetric morbidity/pregnancy in most patients). No language or geographical restriction was applied. Conference abstracts were not excluded.

### Search strategy

A literature search was conducted on the following bibliographic databases: CENTRAL, EMBASE, PubMed, and Web of Science. The search strategies were drafted by two authors and refined by an experienced librarian. The final search strategies for each database are presented in **Supplementary Table 1**. The search in each database was performed from inception. A first literature search was performed on August 20, 2020, followed by an update on January 30, 2022. In addition, we screened the references of retrieved articles for potentially relevant publications.

### Study selection and data extraction

After duplicates removal, two reviewers independently evaluated the titles and abstracts of retrieved publications and, subsequently, the full text of selected articles. Disagreements on study selection were resolved by consensus.

Two reviewers independently extracted data from each of the included primary studies using a prespecified form. Differences were settled through an assessment conducted by a third reviewer. Authors of individual studies were contacted for clarification when needed. Data was extracted on the following: article characteristics (authors, year of publication, country of origin), study aims/purpose, study population, methodology, number of participants with “non-criteria” and definite APS, reported clinical manifestations, treatments, and outcomes. Clinical manifestations were grouped into vascular thrombosis (i.e., arterial, venous, or both) and obstetric morbidity (i.e., more than three abortions before 10 weeks of pregnancy, abortion after 10 weeks of pregnancy, and premature birth before 34 weeks of pregnancy). Information on therapies consisted of the use of low-dose aspirin and anticoagulation. Assessed outcomes included thrombosis recurrence and pregnancy outcomes (i.e., foetal loss or live birth) with or without treatment.

In addition to the global comparative analysis between NC-APS and definite APS patients, whenever such data were available, participants were also classified according to the following subsets of “non-criteria” APS (in the subgroup they more closely fitted) based on the nomenclature we previously proposed (24):

- “Seronegative APS”: patients with clinical manifestations fulfilling APS classification criteria, plus the presence of “non-criteria” manifestations, persistently negative aPL, and exclusion of other thrombophilias that justify their whole clinical presentation. Although not included in our proposal, patients without non-criteria manifestations were also included in this subgroup since they are classified as SN-APS in various studies.
- “Clinical non-criteria APS” (CNC-APS): patients with “non-criteria” manifestations, plus aPL positivity fulfilling the APS classification criteria.
- “Incomplete laboratory APS”: patients with clinical manifestations fulfilling APS classification criteria, plus two or more determinations of aCL between the 95th and 99th percentiles (or positive aCL determinations according to the commercial kit used but below 40 GPL or MPL), and/or two or more determinations of anti-β2GPI antibodies between the 95th and 99th percentiles (low titre patients).
- “Laboratory non-criteria APS” (LNC-APS): patients with clinical manifestations fulfilling APS classification criteria, negative or low titre classification criteria aPL, and positive “non-criteria” aPL testing.
- “Single-positive APS” (SP-APS): Although excluded from our nomenclature proposal, we included this additional group of patients frequently present in the literature to allow for the appraisal of the largest amount of available evidence. This subset includes patients with clinical manifestations fulfilling APS classification criteria and only one single positive determination of criteria aPL.

### Quality assessment

The studies included in the systematic review were evaluated for their risk of bias by using the Newcastle-Ottawa scale (NOS) (25), which, for cohort studies, consists of three parameters (with a total of 8 subitems) - selection of study groups, comparability of groups, and the ascertainment of outcome. The maximum score for these three subsets is 9. Two reviewers independently graded the studies, and any differences were settled through an assessment done by a third reviewer.

## Results

### Search results

**Figure 1:**
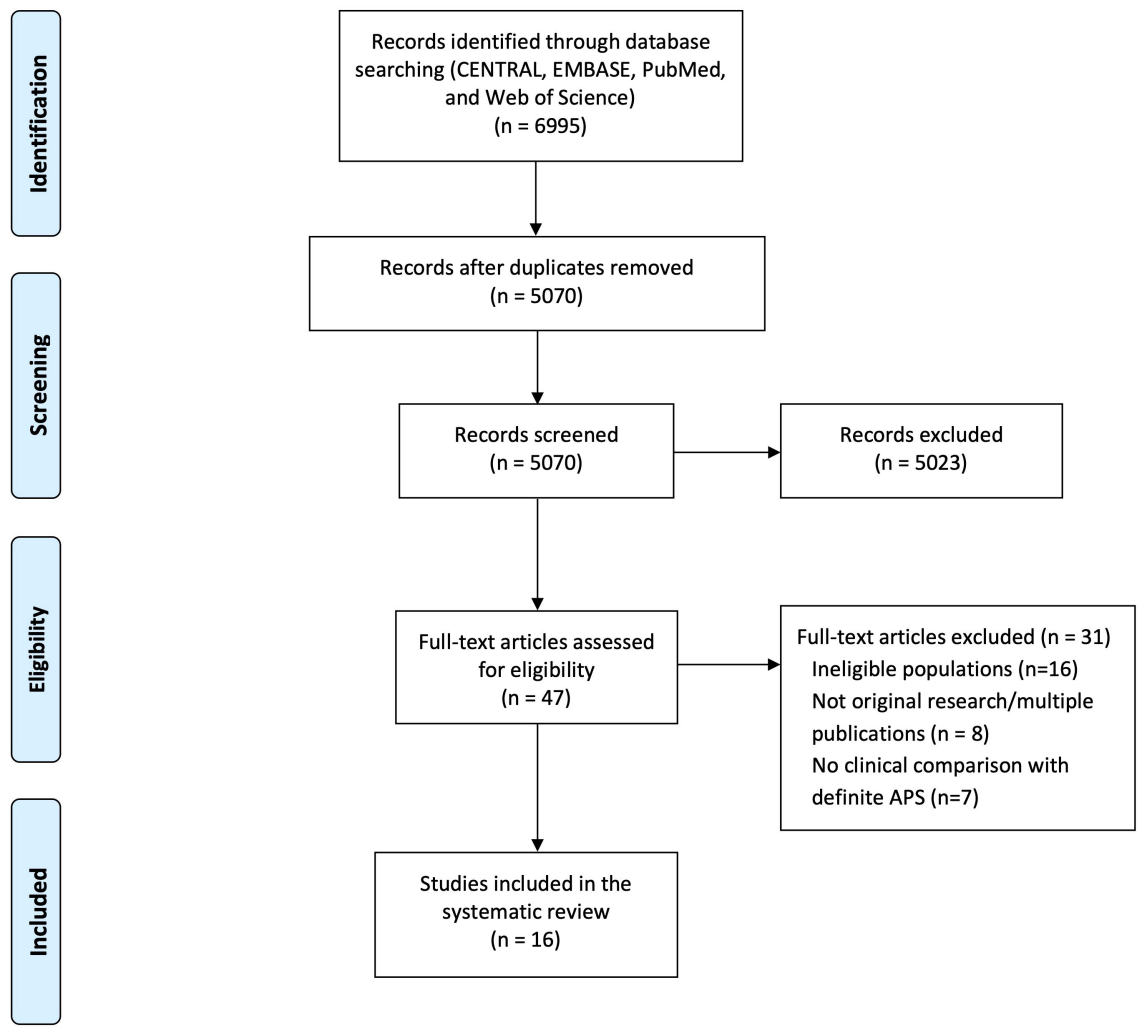
PRISMA flow diagram for the systematic review, detailing database searches, number of abstracts screened, and full texts retrieved.

Initial database search yielded 6,995 references. After duplicates removal, 5,070 citations were identified. Based on their titles and abstracts, 5,023 records were excluded, and 47 full-text articles were retrieved and assessed for eligibility. Of these, 31 were excluded for the following reasons: 16 assessed patients who did not meet the eligibility criteria, eight presented data already described in other publications, while seven described “non-criteria” APS patients but did not compare them with patients with definite APS. The remaining 16 studies were included in the review (**Figure 1**) (4, 12, 26–39), of which one was a conference abstract (36) and the remainder full papers. In ten of the included articles, the specific comparison of outcomes between non-criteria and definite APS was the main objective (4, 26, 30–34, 36–38).

### Description of included studies

The included studies were published between 2012 and 2021 and assessed a total of 3,798 participants (range 36-1,640 per study) (4, 12, 26–39), with 96.7% of female patients. Two studies derive from multicentric European registries (26, 38), and the remainder were performed in eight different countries. Most studies originated from Western Europe (4, 12, 26–29, 32, 33, 38), followed by Asia (30, 31, 37, 39), North Africa (35), the Middle East (34) and South America (36). Consecutive sampling was used in most studies (n=11) (4, 12, 27–30, 32–35, 37), while one study used a convenience sample (26), and three did not specifically state their sampling method (31, 36, 39). Thirteen studies provided information on the comparison between the clinical manifestations of non-criteria and definite APS (4, 12, 26–28, 30, 32–36, 38, 39), while nine studies compared outcomes and therapies used (4, 26, 29–33, 37, 38). A summarized description of included studies is presented in **Table 1**, and a more detailed description of each study´s aims and population are provided in **Supplementary Table 2**. Regarding quality assessment, the mean NOS score value for the 16 included studies was 6.8, with the detailed results available in **Table 2**.

**Table 1:** Description of studies included in the systematic review by country, methodology and number of patients in each group.

Author, year (reference)	Country of origin	Methodology	Number of Patients						
			Definite APS	NC-APS(global)	SN-APS	CNC- APS	Incomplete APS	LNC-APS	Single positive APS
Mekinian, 2012 (32)	France	Retrospective	25	53	21	–	32	–	–
Rodriguez-Garcia, 2012 (4)	United Kingdom	Retrospective	87	67	67	–	–	–	–
Conti, 2014 (12)	Italy	Retrospective	25	24	24	–	–	–	–
Ofer-Shiber, 2015 (34)	Israel	Retrospective	126	117	–	–	117	–	–
Mekinian, 2016 (33)	France	Prospective	83	96	31	–	–	65	–
Omar, 2018 (35)	Egypt	Retrospective	30	30	30	–	–	–	–
Signorelli, 2017 (36)	Brazil	Retrospective	77	13	–	–	–	–	13
Fredi, 2018 (29)	Italy/France (3 centers)	Retrospective	85	81	–	81	–	–	–
Litvinova, 2018 (28)	France	Prospective	41	17	17	–	–	–	–
Shi, 2018 (39)	China	Retrospective	186	48	48	–	–	–	–
Alijotas-Reig, 2019 (26)	30 centers in 10 European countries	Retrospective and Prospective	1000	640	–	289	175^1^	–	175^1^
Abisror, 2020 (38)	14 centers in 5 European countries	Retrospective	285	187	–	–	–	187	–
Ferreira, 2020 (27)	France (3 centers)	Prospective	15^1^	21	21	–	–	–	–
Li, 2020 (30)	China	Prospective	34	94	–	94^1^	94^1^	–	94^1^
Lo, 2020 (31)	Taiwan	Retrospective	12	24	–	–	–	17	7
Yang, 2021 (37)	China	Retrospective	56	32	–	–	–	–	–

^1^NC-APS group includes mixed patients from various subsets

APS, Antiphospholipid Syndrome; CNC-APS, Clinical non-criteria APS; LNC-APS, Laboratory non-criteria APS; NC-APS, Non-criteria antiphospholipid syndrome; SN-APS, Seronegative Antiphospholipid Syndrome.

^1^Only triple-positive APS patients.

**Table 2:** Quality assessment of included studies by the Newcastle-Ottawa Scale.

Author, year (reference)	Selection (0 to 4)	Comparability (0 to 2)	Outcome (0 to 3)	Total (0 to 9)
Abisror, 2020 (38)	3	0	3	6
Alijotas-Reig, 2019 (26)	4	0	3	7
Conti, 2014 (12)	4	0	3	7
Ferreira, 2020 (27)	3	0	3	6
Fredi, 2018 (29)	4	0	3	7
Litvinova, 2018 (28)	4	0	3	7
Li, 2020 (30)	4	0	3	7
Lo, 2020 (31)	3	0	3	6
Mekinian, 2012 (32)	4	0	3	7
Mekinian, 2016 (33)	4	0	3	7
Ofer-Shiber, 2015 (34)	4	0	3	7
Omar, 2018 (35)	4	1	3	8
Rodriguez-Garcia, 2012 (4)	4	1	3	8
Signorelli, 2017 (36)	2	0	3	5
Shi, 2018 (39)	3	0	3	6
Yang, 2021 (37)	4	0	3	7

### Comparison between the clinical manifestations of non-criteria and definite APS

#### Vascular thrombosis

Twelve studies reported on the prevalence of vascular thrombosis in non-criteria APS versus definite APS. Two limited to the description of the prevalence in each group (12, 28), while 10 performed a statistical comparison between groups. The vast majority (7 studies) found no significant difference in the prevalence of thrombosis between NC-APS versus definite APS (1,088 participants) (4, 30, 33–36, 39). Three studies reported vascular events as more common in definite APS (596 participants) (27, 37, 38), but in one case the definite APS group comprised only triple-positive individuals (27) and in another study the authors discuss a possible selection bias due to the employed inclusion criteria (38). No studies reported thrombosis as more frequent in NC-APS. These findings were globally maintained when evaluating specifically venous or arterial thrombosis, and concerned specially the seronegative (5 studies), laboratory non-criteria (3 studies) and incomplete laboratory (2 studies) subgroups of NC-APS. Regarding thrombosis recurrence, two studies analysing SN-APS patients found no significant difference in comparison to definite APS, even though the duration of follow-up was not reported (4, 35). A summary of these results is available in **Table 3**.

**Table 3:** Summary of the main statistical comparisons of the included studies^1^.

Study	Mekinian,2012 (32)	Rodriguez-Garcia,2012 (4)	Ofer-Shiber,2015 (34)	Mekinian,2016 (33)	Omar,2018 (35)	Signorelli,2017 (36)	Fredi, 2018 (29)	Shi, 2018 (39)	Alijotas-Reig, 2019 (26)	Ferreira, 2020 (27)	Li, 2020 (30)	Lo, 2020 (31)	Abisror, 2020 (38)	Yang, 2021 (37)
Type of NC-APS	IncompleteAPSSN-APS	SN-APS	IncompleteAPS	LNC-APSSN-APS	SN-APS	Single-positive	CNCAPS	SN-APS	Incomplete APSCNC-APSSingle-positive	SN-APS	Incomplete APSLNC-APSSingle-positive	LNC-APSSingle-positive	LNCAPS	Expert consensus^2^
Parameter														
**Vascular thrombosis**										^3^				
***Venous thrombosis***								^4^						
***Arterial thrombosis***								^4^						
**Thrombosis recurrence**														
**Obstetric morbidity**														
***≥3 spontaneous abortion <10w***														
***Fetal loss >10w***														
***Prematurity <34w***														
**Treatment frequency** **(thrombotic APS)**														
**Treatment frequency** **(obstetric APS)**							^5^						^6^	
**Pregnancy outcomes (obstetric APS)**									^7^			^8^		
												^9^		

^1^Yellow boxes represent studies where no differences were found in the evaluated outcome between NC-APS and definite APS patients; green boxes those where the outcome was more frequent in definite APS; red those where the outcome was more frequent in NC-APS; grey boxes represent studies where the specific variable was not evaluated.

^2^Definition according to the Expert consensus on diagnosis and management of obstetric antiphospholipid syndrome of the Chinese Medical Association Society of Perinatal Medicine. The full-text article with the exact definition was not obtainable.

^3^Only triple-positive individuals in the definite APS group.

^4^But simultaneous presence of arterial and venous thrombosis was more common in SN-APS (p=0.012).

^5^Higher use of aspirin/LMW combination in definite APS.

^6^No difference in the frequency of treated pregnancies and use of aspirin/LMWH combination, but higher rate of therapeutic LMWH dose use in definite APS and of aspirin monotherapy in SN-APS.

^7^Live birth rate was similar, but more pregnancy complication occurred in NC-APS (p<0.001).

^8^LNC-APS.

^9^Incomplete laboratory APS.

APS, Antiphospholipid Syndrome; LMWH, Low-molecular-weight heparin; NC-APS, Non-criteria antiphospholipid syndrome.

#### Obstetric morbidity

Fourteen studies compared the prevalence of obstetric morbidity in NC-APS versus definite APS. Two studies solely described the prevalence in each group (12, 28), while 12 performed a statistical comparison. The majority of these studies (seven) found no statistical differences in the global prevalence of pregnancy morbidity (970 participants) (4, 29, 32–36), 3 studies found obstetric manifestations as more common in definite APS than in NC-APS (1,96*2* participants) (26, 37, 39), while the opposite occurred in 2 studies (508 participants) (27, 38). These results were globally maintained when evaluating specific Sydney criteria manifestations (i.e., three or more spontaneous abortions before 10 weeks of gestation, fetal death, and prematurity before 34 weeks of gestation). In a large European registry on obstetric APS (EUROAPS) (26), a noticeable difference between these groups was the chronology of placental vasculopathy – predominantly prior to 34 weeks in definite APS and subsequent to 34 weeks in NC-APS patients. These studies evaluating obstetric morbidity encompassed the seronegative (6 studies), incomplete laboratory (3 studies), laboratory non-criteria, clinical non-criteria, and single-positive (2 studies each) subgroups of NC-APS. A summary of these results is available in **Table 3**.

### Comparison between the treatment and outcomes of “non-criteria” and definite APS

#### Treatment frequency and regimens

Only one study compared the treatment frequency of thrombotic NC-APS (specifically SN-APS) and definite APS patients, with no significant difference in the percentage of patients under anticoagulation between groups (59.6% for SN-APS versus 60.8% for definite APS) (4).

Concerning obstetric APS, 9 studies reported a comparison of the treatment frequency and regimens in non-criteria versus definite APS, with all but one describing similar treatment frequency (2,762 participants) (4, 26, 29–32, 37, 38), and one reporting more frequent treatment in definite obstetric APS (179 participants) (33). Of note, the aspirin dosage was not discriminated in 5 studies (4, 31, 37, 38), it was referred as “low-dose aspirin” in two studies (26, 29), and specified as 100 mg/day in one study (32) and 50-75 mg/day in another (30). In two of the studies the use of aspirin/LMWH was more common in definite APS (29, 33), and in another the study this was the case for the use of therapeutic dose of LMWH (38). The reported percentage of NC-APS patients submitted to treatment during pregnancy ranges from 26% to 100% (4, 26, 29–33, 37, 38), but apart from the study with the lowest use (33), all others state percentages above 75%. Studies evaluating treatment frequency covered the laboratory non-criteria (4 studies), seronegative, single-positive, and incomplete laboratory (3 studies each), and clinical non-criteria (2 studies) subgroups of NC-APS. Detailed results are presented in **Table 3**.

#### Treatment and pregnancy outcomes

In the field of pregnancy morbidity, in the 10 studies reporting a statistical comparison of the pregnancy outcomes of NC-APS versus definite APS, 7 found similar outcomes, including successful pregnancies/live births (1,171 participants) (29, 30, 32, 33, 35, 37, 38). The remaining three (1,830 participants) (4, 26, 31) even described worse outcomes/increased complications at least in some subset of NC-APS in comparison with definite APS: in one the rate of successful pregnancies was lower in women with SN-APS (38.2% vs 50.2%) (4); in a large European registry, even though the rate of live births were similar, obstetric complications occurred in 470 of 640 pregnancies (73.4%) in NC-APS and in 651 of 1000 pregnancies (65.1%) in definite APS (26); and in a study evaluating patients with non-criteria aPL (AhPL isotypes) live births occurred in 53.9% of patients versus 100% in definite APS patients, even though they all were submitted to treatment (31). These studies evaluating pregnancy outcomes covered the laboratory non-criteria and seronegative (4 studies each), single-positive, and incomplete laboratory (3 studies each), and clinical non-criteria (2 studies) subgroups of NC-APS. A summary of these results is available in **Table 3**.

Regarding the effects of treatment on pregnancy outcomes, 5 studies described an improvement of live births in both NC-APS and definite APS with treatment (26, 32, 33, 37, 38), including patients of the laboratory non-criteria (3 studies), seronegative and incomplete laboratory (2 studies each), and single-positive (1 study) subgroups of NC-APS. In a large European retrospective study analysing LNC-APS patients, the cumulative incidence of adverse obstetrical events was significantly improved in treated patients versus untreated ones, but no difference was found between those receiving aspirin or aspirin/LMWH combination (38). Another study devoted to LNC-APS patients also revealed this finding, with aspirin/LMWH combination being used less frequently in NC-APS, whereas the number of pregnancies with favourable outcome was similar to that of definite APS (33). This lack of difference in pregnancy outcomes between women treated with combination therapy and those receiving aspirin monotherapy was also present in a study including CNC-APS patients (29).

## Discussion

This systematic review points towards the absence of marked differences between non-criteria and definite APS in most of the evaluated parameters related to clinical manifestations, therapy, and clinical outcomes.

Regarding thrombotic manifestations, the majority of studies found no significant difference regarding the prevalence of arterial/venous thrombosis or thrombosis recurrence. Studies evaluating the occurrence of thrombosis were conducted mainly on seronegative and laboratory non-criteria patients. Conversely, most obstetric studies included an incomplete laboratory APS group (i.e., low titre aPL). This reinforces the previously described notion that low aPL titres, such as those seen in incomplete laboratory APS patients, seem to be particularly implicated in pregnancy morbidity rather than in vascular thrombosis (40, 41). In the specific case of SN-APS, there was the additional suggestion of no difference in thrombosis recurrence.

Regarding obstetric manifestations, most studies also displayed no significant difference in their prevalence between definite and NC-APS; the fact that most studies, including those with most participants (26, 38), focus mainly on obstetric NC-APS may hint a predominantly obstetric phenotype in non-criteria patients.

The review on treatment regimens revealed, firstly, the paucity of data on thrombotic APS, with practically all studies focusing on obstetric patients. An interesting finding was the fact that, despite the absence of formal recommendations, many obstetric NC-APS patients are already treated in a similar fashion to those with definite APS, with no significant difference found in the global prevalence of pregnancy treatment in nearly all studies. This may reflect either the tendency of clinicians to offer treatment when faced with adverse pregnancy outcomes or the eagerness of patients to take them despite the unproven benefits (22). These situations have been reported to thwart the development of clinical trials involving these patients (42). Nevertheless, a significant number of studies described an improvement in live births after treatment in NC-APS, with three studies additionally signalling the possibility that aspirin monotherapy might be as efficacious as aspirin/LMWH combination in these patients (29, 33, 38). This information adds further data to the notion that there may be benefit in treating at least some subgroups of these non-criteria patients, as supported by a recent study displaying improved outcomes with treatment in low titre patients (43). This is a relevant finding, as evidence in this matter is scarce and previous works from an Italian group found no improvement in pregnancy outcomes with the use of low-dose aspirin in patients with “incomplete” obstetric APS (44, 45). Nevertheless, attention should be given to the fact that, in the included articles, there are variations in the treatments prescribed between different institutions, patient groups (i.e., definite and NC-APS) and even among patients of the same group, partially hampering the analysis of the value of specific therapies and regimens.

The noticeable absence of differences in most studies/parameters between definite and NC-APS could underscore the need for a more active evaluation and follow-up of these individuals as, on many occasions, since they do not fulfil the criteria, are not referred for evaluation, or are assessed and discharged without further surveillance. It also highlights the need to further study these individuals to identify in which subsets of non-criteria patients these similarities are effectively present.

This systematic review has strengths but also significant weaknesses. The low-quality evidence (i.e., heterogeneous study populations and designs) and the fact that many studies did not focus on the research questions as their main objective are important limitations. A fact that may affect the frequency of the different parameters evaluated across the studies is the variable definition of non-criteria patients. For instance, studies that do not exclude the presence of other thrombophilia in NC-APS may overestimate the presence of events in these patients, as they may be related to any of these untested thrombophilia; studies which require the presence of non-criteria manifestations in NC-APS patients may also increase the prevalence of thrombosis in these patients, as some of these features are linked with an increased risk of events (46); and studies which include patients with incomplete laboratory criteria (i.e., low titre or single positive aPL) could hypothetically display higher prevalence of events than seronegative patients. In the case of the global proportion of thrombosis/obstetric patients, differences may be consequential to the recruitment site (e.g., thrombosis clinic, pregnancy clinic) resulting in a higher proportion of recruited obstetric or thrombosis patients. Additionally, the fact that only observational studies with considerable risk of bias were included, particularly in the comparability between groups item of the NOS, demonstrates the lack of high-quality research in this field and calls for caution in the interpretation and extrapolation of the results of the retrieved articles. Furthermore, the absence of appropriate matching in the control group of some articles in variables such as age, gender and type of APS (i.e., obstetric or thrombotic) may add possible confounders and further undermine the comparison between groups. A curious finding is the fact that two of the studies with larger samples (26, 38) displayed more prominent differences in outcomes between groups, what could lead to the idea that with a higher number of participants, more disparities could be found between non-criteria and definite APS. Nevertheless, these were also two studies flagged with a considerable risk of bias. One (26) features, in its inclusion criteria for the NC-APS group, mostly patients with only non-criteria obstetric manifestations - this results automatically in a markedly reduced prevalence of criteria manifestations in comparison with definite APS patients, in contrast with most of the other studies. Additionally, one of the subgroups of patients included in this study features individuals who do not meet clinical nor laboratory criteria for definite APS, a fact that might also partially weaken the comparison between groups. In the second one (38), the authors themselves discuss a possible selection bias due to the employed inclusion criteria, where the need for the presence of at least one pregnancy to be included in the NC-APS group might have significantly undermined the inclusion of thrombotic phenotypes in comparison with the definite APS group.

Despite these limitations, the broad search conducted in various databases with the absence of restrictions in publication date and inclusion of congress abstracts allowed for a comprehensive evidence collection. This deliberately far-reaching strategy was adopted, given the heterogeneity in this subject, to ensure an extensive evaluation of all data available. Additionally, the attempt to compartmentalize NC-APS in different subsets also allowed, in some cases, a clearer analysis of the specific type of patients included in each study.

This systematic review hints that most clinical manifestations (obstetric and vascular/thrombotic) are not markedly different between definite and NC-APS. Furthermore, it suggests that most pregnancies in obstetric NC-APS are already being treated as definite APS, and that pregnancy treatment in NC-APS might carry improved outcomes. Aspirin monotherapy might be sufficient in these patients. These findings imply a potential value in a more active follow-up of these patients and hypothesize a possible benefit in managing at least some subgroups of non-criteria patients in a similar fashion to that of definite APS. Nevertheless, the generalization of these results is undermined by the low-quality evidence available, highlighting the need for well-defined and homogenous NC-APS populations in future studies.

## Data availability statement

The original contributions presented in the study are included in the article/**Supplementary Material**. Further inquiries can be directed to the corresponding author.

## Author contributions

GR participated in the study design, data search and extraction, and data analysis. EF participated in the study design, data search, and extraction. BS-P participated in the study design and data analysis. IR-P, AM, IB, RC, and GE participated in the study design, data search and extractions (as referees). All authors read and approved the final manuscript.

## Funding

This research was funded by the *Grant for Studies in Autoimmunity* of the Autoimmune Diseases Study Group of the Portuguese Society of Internal Medicine.

## Acknowledgments

*In memoriam* of Professor Paulo Bettencourt, highlighting his remarkable clinical career and vital contribution to the works performed by this research group. The authors wish to thank Helena Donato, from the Documentation Unit, Centro Hospitalar e Universitário de Coimbra, Coimbra, Portugal, for her assistance performing the search for the systematic review.

## Conflict of interest

The authors declare that the research was conducted in the absence of any commercial or financial relationships that could be construed as a potential conflict of interest.

## Publisher’s note

All claims expressed in this article are solely those of the authors and do not necessarily represent those of their affiliated organizations, or those of the publisher, the editors and the reviewers. Any product that may be evaluated in this article, or claim that may be made by its manufacturer, is not guaranteed or endorsed by the publisher.
