# Supplementary material for: Comparison of non-criteria antiphospholipid syndrome with definite antiphospholipid syndrome: A systematic review
Source: Front Immunol. 2022 Aug 18;13:967178. doi: 10.3389/fimmu.2022.967178 (PMC9434011; doi:10.3389/fimmu.2022.967178)
Supplement: Supplementary file 1 [file DataSheet_1.pdf]

Supplementary table 1. Search strategies.

**CENTRAL – Cochrane Central Register of Controlled Trials**<br>antiphospholipid syndrome OR Anti-Phospholipid syndrome OR Hughes Syndrome OR Anti Phospholipid Antibody Syndrome OR antibodies antiphospholipid OR Antibody Antiphospholipid in Title Abstract Keyword AND gap OR incomplete OR low titer OR low titre OR non-conventional OR non-criteria OR possible OR probable OR seronegative OR transient OR noncriteria in Title Abstract Keyword - in Trials (Word variations have been searched)
**EMBASE**<br>(gap:ab,ti OR incomplete:ab,ti OR 'low titer':ab,ti OR 'low titre':ab,ti OR 'non conventional':ab,ti OR 'non criteria':ab,ti OR possible:ab,ti OR probable:ab,ti OR seronegative:ab,ti OR transient:ab,ti OR noncriteria:ab,ti) AND ('antiphospholipid syndrome':ab,ti OR 'anti-phospholipid syndrome':ab,ti OR 'hughes syndrome':ab,ti OR 'anti phospholipid antibody syndrome':ab,ti OR 'phospholipid antibody'/exp OR 'antiphospholipid syndrome'/exp)
**Pubmed**<br>(antiphospholipid syndrome OR Anti-Phospholipid syndrome OR Hughes Syndrome OR Anti Phospholipid Antibody Syndrome OR antibodies antiphospholipid OR Antibody Antiphospholipid OR "Antibodies, Antiphospholipid"[Mesh] OR "Antiphospholipid Syndrome"[Mesh]) AND (gap[Title/Abstract] OR incomplete[Title/Abstract] OR low titer[Title/Abstract] OR low titre[Title/Abstract] OR non-conventional[Title/Abstract] OR non-criteria[Title/Abstract] OR possible[Title/Abstract] OR probable[Title/Abstract] OR seronegative[Title/Abstract] OR transient[Title/Abstract] OR noncriteria[Title/Abstract])
**Web of Science**<br>(antiphospholipid syndrome OR Anti-Phospholipid syndrome OR Hughes Syndrome OR Anti Phospholipid Antibody Syndrome OR antibodies antiphospholipid OR Antibody Antiphospholipid OR Antibodies Antiphospholipid OR Antiphospholipid Syndrome) AND (gap OR incomplete OR low titer OR low titre OR non-conventional OR non-criteria OR possible OR probable OR seronegative OR transient OR noncriteria)

Supplementary table 2. Description of Studies Included in the Systematic Review by Aims and Study population

Author, year (reference)	Aims/purpose	Study population
Mekinian, 2012 [33]	Assessment of the association of low-titer aPL with APS-like obstetrical events<br>Analyze the impact of conventional treatment in patients with low titer aPL levels	**25** definite OAPS patients<br>**32** patients with obstetrical events consistent with clinical APS, but with aCL or anti-β2GPI between the 90th and 99th percentile<br>**21** obstetrical APS-like untreated pregnancies, but without aPL
Rodriguez-Garcia, 2012 [4]	Comparison of thrombotic events and pregnancy morbidity between SN-APS and definite APS patients	**87** definite APS patients<br>**67** SN-APS patients with clinical manifestations of APS testing negative for criteria aPL, with the presence of at least two “non-criteria” manifestations
Conti, 2014 [12]	Identification of the best screening combination of “new” antigenic targets or methodological approaches to detect aPL in SN-APS patients	**25** definite APS patients<br>**24** SN-APS patients with clinical features consistent with APS but persistently negative for criteria aPL
Ofer-Shiber, 2015 [35]	Evaluation of APS features in patients tested positive for aPL regardless of the level of aCL and/or anti-β2GPI	**126** definite APS patients with aCL 40 U or above and and/or anti-β2GPI above the 99th percentile (with or without positive LAC)<br>**117** low titer patients (not fulfilling the laboratory criteria above)
Mekinian, 2016 [34]	Comparison of the pregnancy outcome between patients with non-criteria aPL, definite APS, and patients with clinical obstetrical criteria for APS and negative aPL (criteria and non-criteria)	**31** “non-aPL” patients (with criteria obstetric manifestations but negative criteria and non-criteria aPL)<br>**65** patients with non-criteria aPL (“non-conventional APS”)<br>**83** definite APS patients
Omar, 2018 [36]	Evaluation of the clinical usefulness of anti-annexin A5 antibodies in patients with definite APS and SN-APS	**30** definite APS patients<br>**30** SN-APS patients with clinical manifestations of APS testing negative for criteria aPL, with the presence of at least two “non-criteria” manifestations
Signorelli, 2017 [37]	Evaluation of the clinical differences between definite APS and “probable” APS (transient aPL positivity)	**77** definite APS patients<br>**13** patients with APS clinical manifestations but transient aPL (only one detection of criteria aPL after 2 or more assays)
Fredi, 2018 [29]	Evaluation of the risk factors for adverse pregnancy outcomes in patients with confirmed aPL positivity, aPL carriers and primary APS patients.	**85** definite obstetric APS patients with 124 pregnancies<br>**42** definite thrombotic APS patients (with or without pregnancy morbidity) with **66** pregnancies<br>**39** patients with “non-criteria” manifestations and aPL positivity with 54 pregnancies
Litvinova, 2018 [28]	Evaluation of the usefulness of non-conventional APS markers for the diagnosis of patients with clinical manifestations of APS and SN-APS	**41** definite APS patients<br>**17** SN-APS patients (persistent negativity for the conventional antibodies with a strong clinical suspicion of APS)
Alijotas-Reig, 2019 [26]	Comparison of the clinical features, laboratory data and fetal-maternal outcomes between patients with OAPS and patients with aPL-related obstetric complications not fulfilling Sydney criteria	**1000** definite OAPS patients and **640** NC-OAPS patients - 3 subgroups:<br>Subgroup A: 176 women with OMAPS and medium/low aPL titers<br>Subgroup B: 175 women with Sidney clinical criteria and medium/low aPL titers or intermittent positivity for one aPL<br>Subgroup C: 289 women with OMAPS and fulfilling Sydney laboratory criteria
Ferreira, 2020 [27]	Evaluation of the value of annexin-A5 anticoagulant ratio and non-criteria antibodies for the diagnosis of APS in patients with clinical seronegative APS	**15** definite APS triple-positive patients<br>**21** SNAPS patients (clinical manifestations of APS according to Sydney criteria but testing negative for aPL)
Abisror, 2020 (12)	Comparison of clinical characteristics and pregnancy treatments of seronegative and seropositive APS	**187** patients with non-criteria aPL (“seronegative APS”)<br>**285** definite APS patients
Liu, 2020 [31]	Evaluation of the value of non-criteria antibodies to assist in the detection of SN-APS patients and their value in predicting adverse clinical events	**192** definite APS patients<br>**90** SN-APS patients (fulfilling Sydney clinical criteria but persistently negative for criteria aPL). Hereditary and other acquired thrombophilia were excluded, and had at least one of the non-criteria manifestations associated with APS
Li, 2020 [30]	Comparison of differences between criteria OAPS and NC-OAPS patients	**34** definite obstetric APS patients<br>**94** NC-OAPS patients<br>NC-OAPS included: (1) 1 time or intermittently positive for aCL or/and aβ2GPI (> 95th percentile) or/and LA with ≥ 2 times early fetal loss, late fetal loss or adverse pregnancy outcomes due to pre-eclampsia, eclampsia, placental abruption or IUGR; (2) ≥ 2 times positive for aCL or/and aβ2GPI (95th–99th percentile) with early or late fetal loss or adverse pregnancy outcomes due to pre-eclampsia, eclampsia, placental abruption or IUGR; (3) ≥ 2 times positive for aCL or/and aβ2GPI (≥ 99th percentile) or/and LA with one or two times early fetal loss
Lo, 2020 [32]	Evaluation of the pregnancy outcomes for women with non-criteria antiphospholipid syndrome after anticoagulant therapy	**12** OAPS patients<br>**7** Suspected obstetric APS patients: obstetric clinical criteria plus criteria laboratory test (positive only once)

		**17** Non-criteria obstetric APS patients: APS clinical criteria, persistent negative results for criteria laboratory tests and positive non-criteria laboratory test
Yang, 2021 (15)	Evaluation of clinical characteristicas and pregnancy outcomes of NC-OAPS patients under anticoagulation	**56** OAPS patients<br>**32** NC-OAPS patients according to the Expert consensus on diagnosis and management of obstetric antiphospholipid syndrome of the Chinese Medical Association Society of Perinatal Medicine

Abbreviations – aPL: Antiphospholipid antibodies; APS: Antiphospholipid Syndrome; NA: Non-available/non-applicable; NC-APS: Non-criteria antiphospholipid syndrome; OAPS: Obstetric antiphospholipid syndrome; SN-APS: Seronegative Antiphospholipid Syndrome.
